# Antibacterial Activity of Cissus quadrangularis (Veldt Grape) Against Porphyromonas gingivalis, a Keystone Pathogen in Periodontal Disease: An In Vitro Study

**DOI:** 10.7759/cureus.66377

**Published:** 2024-08-07

**Authors:** Aathira R Nair, Prem Blaisie Rajula, Ravishankar PL, Sai Sri Soury Geddam, Subhashini B, Lochini S

**Affiliations:** 1 Periodontology, SRM Kattankulathur Dental College and Hospital, Chennai, IND

**Keywords:** porphyromonas gingivalis, minimum inhibitory concentration, ethanolic extract, cissus quadrangularis, antibacterial activity

## Abstract

Background: *Porphyromonas gingivalis*, a keystone pathogen and one of the primary pathogens responsible for periodontitis, leads to a chronic inflammatory condition that destroys the periodontal tissues and ultimately results in tooth loss. While conventional non-surgical therapy combined with antibiotics and local drug delivery systems are commonly used to treat periodontitis, certain medicinal herbs have also demonstrated efficacy in its prevention. *Cissus quadrangularis* L. (CQ), a perennial plant from the Vitaceae family, is widely recognized and used as a medicinal herb in many tropical countries, predominantly in India, Sri Lanka, Thailand, Java, West Africa, and the Philippines.

Aim: The aim of the study was to determine the antibacterial activity of CQ against the periodontal keystone pathogen *P. gingivalis*.

Method: Aqueous and ethanolic extracts of CQ were prepared using a Soxhlet extractor. The antibacterial effectiveness of these extracts against the periodontal pathogen *P. gingivalis* was evaluated at different concentrations, and the minimal inhibitory concentration (MIC) was determined using broth microdilution.

Results: The ethanolic extract of CQ mixed with 10% dimethyl sulfoxide (DMSO) showed higher inhibition compared to the aqueous extract of CQ against *P. gingivalis*.

Conclusion: Our study revealed the potent inhibitory effects of CQ against *P. gingivalis*. Both aqueous and ethanolic extracts displayed MIC values of 500 µg/mL. Notably, the ethanolic extract of CQ, dissolved in 10% DMSO, demonstrated superior efficacy with a lower IC50 value of 194.36 µg/mL. These findings indicate promising potential for CQ in the management of periodontal disease.

## Introduction

The keystone pathogen *Porphyromonas gingivalis* is prominently implicated in periodontitis, a chronic inflammatory condition that results in tooth loss and the damage of gingiva, alveolar bone, and periodontal ligaments [1]. Among the myriad bacterial species inhabiting the oral cavity, this specific gram-negative rod bacterium is non-motile and requires anaerobic conditions to survive. After 3-5 days of incubation, it forms black-pigmented colonies on supplemented blood agar plates [2,3]. By interacting with commensal *Streptococci*, *P. gingivalis* causes secondary infections in periodontal tissues [4]. Its capacity to elude host immune responses and disseminate via cell-to-cell interactions or hematogenous routes sustains persistent irritation in the peripheral regions [5].

Presently, periodontitis management involves systemic and localized antibiotic therapy, sometimes coupled with surgical interventions for addressing deep-pocket irritations [6]. Anaerobic bacteria, especially *P. gingivalis*, are the target of antibiotics. A range of antibiotics such as metronidazole, amoxicillin, amoxicillin/clavulanic acid, clindamycin, tetracycline, and fluoroquinolones are utilized alongside mechanical debridement [7,8].

Failure to perform debridement can lead to antibiotics being unable to penetrate biofilm, resulting in significantly higher minimal inhibitory concentrations (MIC) [9]. Moreover, *P. gingivalis* strains exhibit considerable resistance rates, reaching up to 21.56% for metronidazole, 25.49% for amoxicillin, and 23.52% for clindamycin [10]. Notable resistance is also observed for penicillin, erythromycin, azithromycin, and tetracycline [11].

As antibiotic resistance escalates, the imperative for new antimicrobials intensifies, yet the rate of new antibiotic discoveries has declined [12]. Medicinal plants offer another avenue for combating *P. gingivalis *infections. Not only do plant species represent a diverse reservoir of antibacterial compounds [13], but ethnobotanical knowledge can guide the selection of plant extracts for discovering novel antibacterials [14]. Extensive ethnobotanical documentation exists regarding plant usage for oral health, yet further research is assured to establish the pharmacological efficacy of these ethnomedicinal practices against dental pathogens.

**Figure 1:**
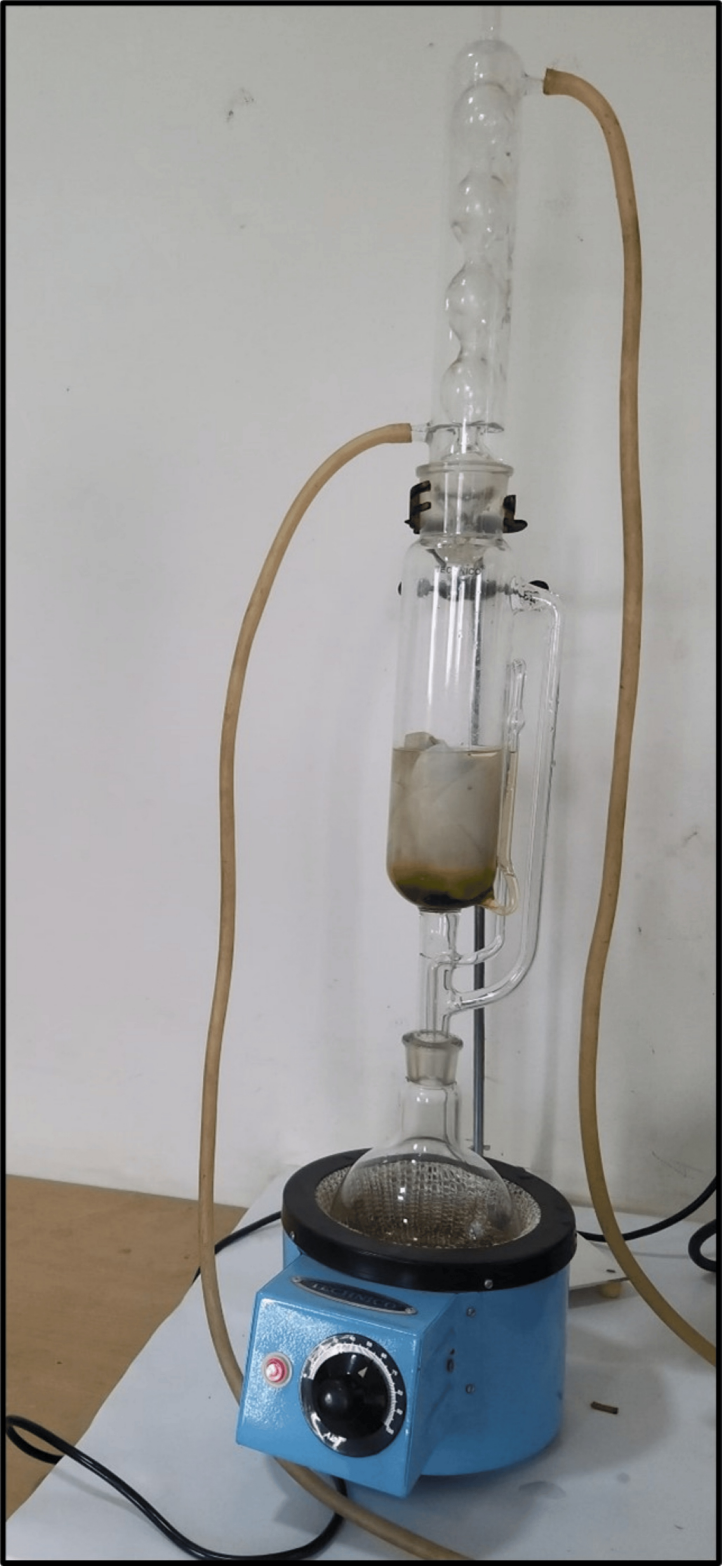
Extract preparation using a Soxhlet apparatus

*Cissus quadrangularis* (CQ), also known as Veldt Grape or Devil's Backbone, belongs to the Vitaceae family. It is recognized by various names, including Asthisamharaka, Hadjod, Pirandai, Mangara Valli, and *Vitis quadrangularis *[15-19]. Utilized in Ayurvedic and Siddha medicine traditions, it offers a range of therapeutic benefits, including antimicrobial, antiulcer, antioxidative, anti-osteoporotic, gastroprotective, cholinergic, antihyperglycemic, and cardiovascular properties [19]. This cactus-like plant is characterized by jointed climbers with simple tendrils and leaves that are either simple or lobed, cordate, serrate, or dentate. Its small, greenish-white, bisexual, and tetramerous flowers complement its versatile medicinal properties (Figure 1). This plant possesses the capability to mend bones, earning it the epithet "bone setter" [20].

It was discovered to exhibit anti-osteoporotic, antioxidant, antimicrobial, anticonvulsant, sedative, neuropharmacological, central nervous system (CNS) activity, anthelmintic, antidiabetic, antistress, anticancerous potential, and antinociceptive properties [18,21].

Previous research has revealed varying degrees of antibacterial efficacy against different microorganisms [16], yet scant literature has examined its antibacterial activity specifically against *P. gingivalis*. Therefore, this study aimed to assess the antibacterial potential of CQ against this significant periodontal pathogen.

## Materials and methods

Collection of sample

Commercially available CQ powder (Bixa Botanicals, Dhar, India), also known as Hadjod stem powder, was used.

Preparation of ethanolic extract of CQ* *(Soxhlet extraction method)

**Figure 2:**
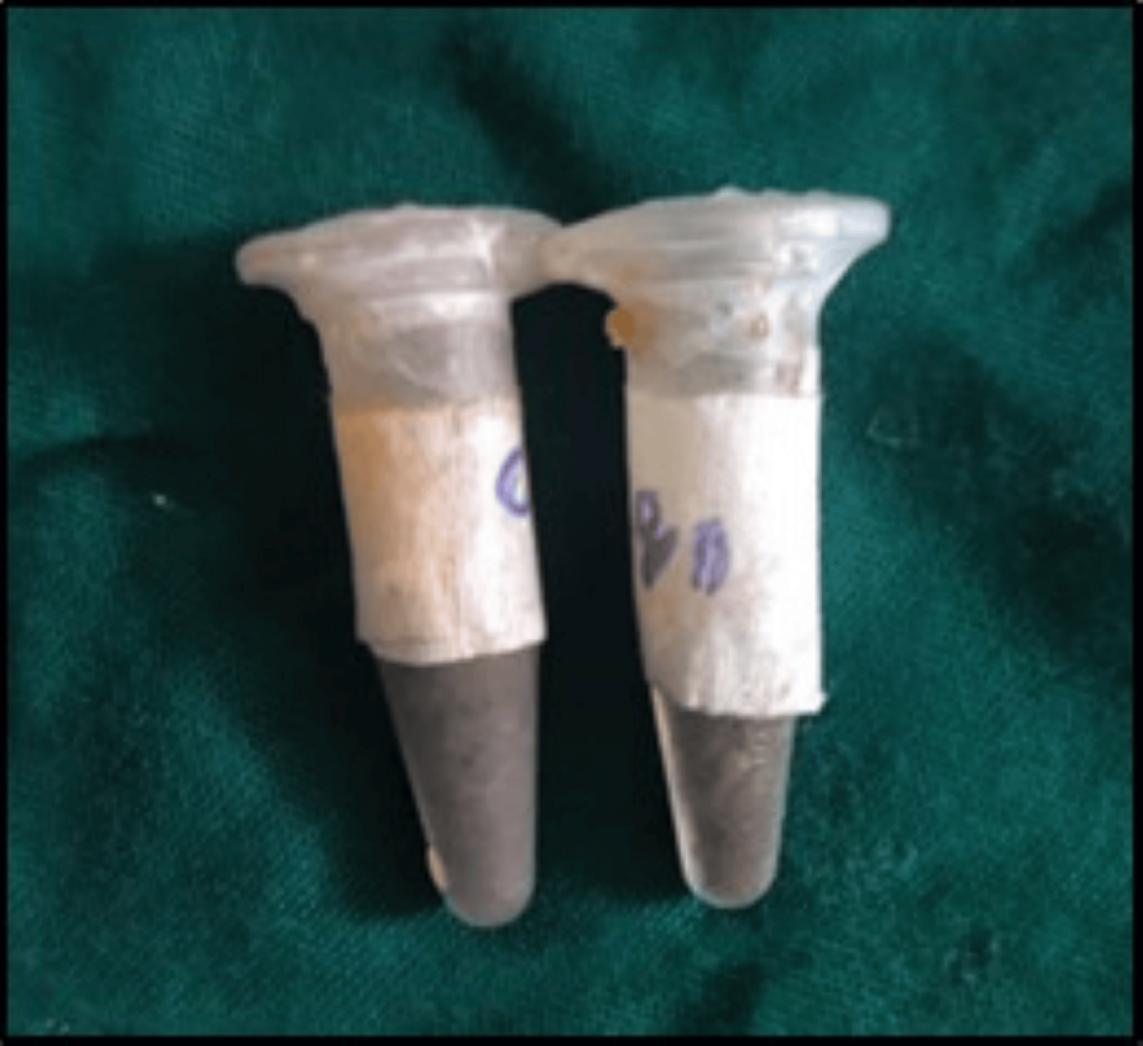
Aqueous and ethanolic extract of Cissus quadrangularis

Ethanolic extract of CQ was prepared using a Soxhlet extractor (Figure 1). Among conventional methods, Soxhlet was the most powerful method and had the most extraction yields [22]. The solvent (250 mL ethanol) was filled in the percolator. Finely ground powder of CQ (25 g) was packed using Whatman filter paper and placed inside the thimble. The solvent (ethanol) was heated by adjusting the temperature of the mantle to 50°C to reflux. The solvent vapor that travels up the distillation arm was cooled by the water that circulates in the condenser. The active compounds of CQ were extracted in ethanol. The cycle was repeated thrice to enable the complete extraction of the bioactive compounds present in CQ. After extraction, the solvent was evaporated to yield the extracted compounds (Figure 2). The ethanolic extract of CQwas dissolved in 10% dimethyl sulfoxide (DMSO) at a concentration of 25 mg/mL. Similarly, the aqueous extract was also obtained using the Soxhlet extraction method.

Preparation of bacterial suspension

From the maintained frozen stock cultures of *P. gingivalis* (ATCC No-33277), a small quantity of cells was recovered and subcultured. Brain heart infusion (BHI) broth was used as the culture medium to support the growth of bacteria. This culture was transferred into tubes containing 2 mL of the BHI medium to obtain a culture suspension of *P. gingivalis*. The selected test bacterial strain was adjusted to 0.5 McFarland turbidity standards.

MIC by broth dilution method

**Figure 3:**
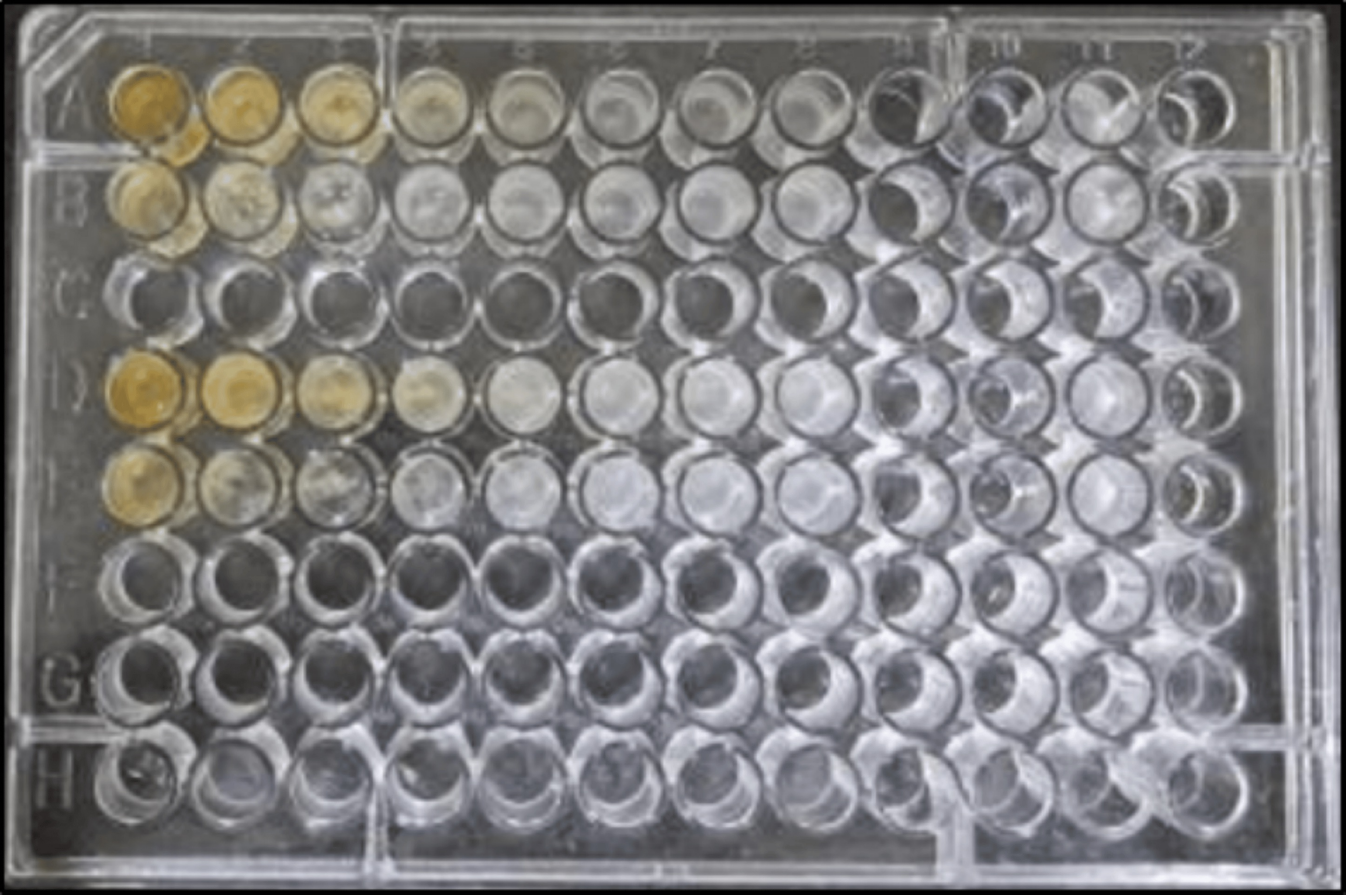
Microdilution. Rows A and D: aqueous extract of CQ. Rows B and E: ethanolic extract of CQ dissolved in 10% DMSO. CQ: *Cissus quadrangularis* L., DMSO: dimethyl sulfoxide.

The MIC was calculated using the broth microdilution method with a 96-well polystyrene flat bottom plate. From columns 2 to 8, 50 μL of Mueller Hinton broth (MHB) was added to the microtiter plate. Then, 100 μL of the sample was added to the first well and serially diluted to the eighth well (1000 μg/mL to 7.8 μg/mL) (rows A and D: aqueous extract of CQ; rows B and E: ethanolic extract of CQdissolved in 10% DMSO). The 10th and 11th wells served as sterility and negative controls. Then, 50 μL of the *P. gingivalis* (ATCC® 33277) suspensions (0.5 McFarland) was added to all columns except the sterility column (Figure 3). The 96-well plate was read at 600 nm after being incubated for 24 hours at 37 °C. MIC was determined by comparing the growth rate of the tested bacterial strain with the negative controls. The lowest extract concentration that inhibited bacterial growth was noted as MIC.

## Results

**Figure 4:**
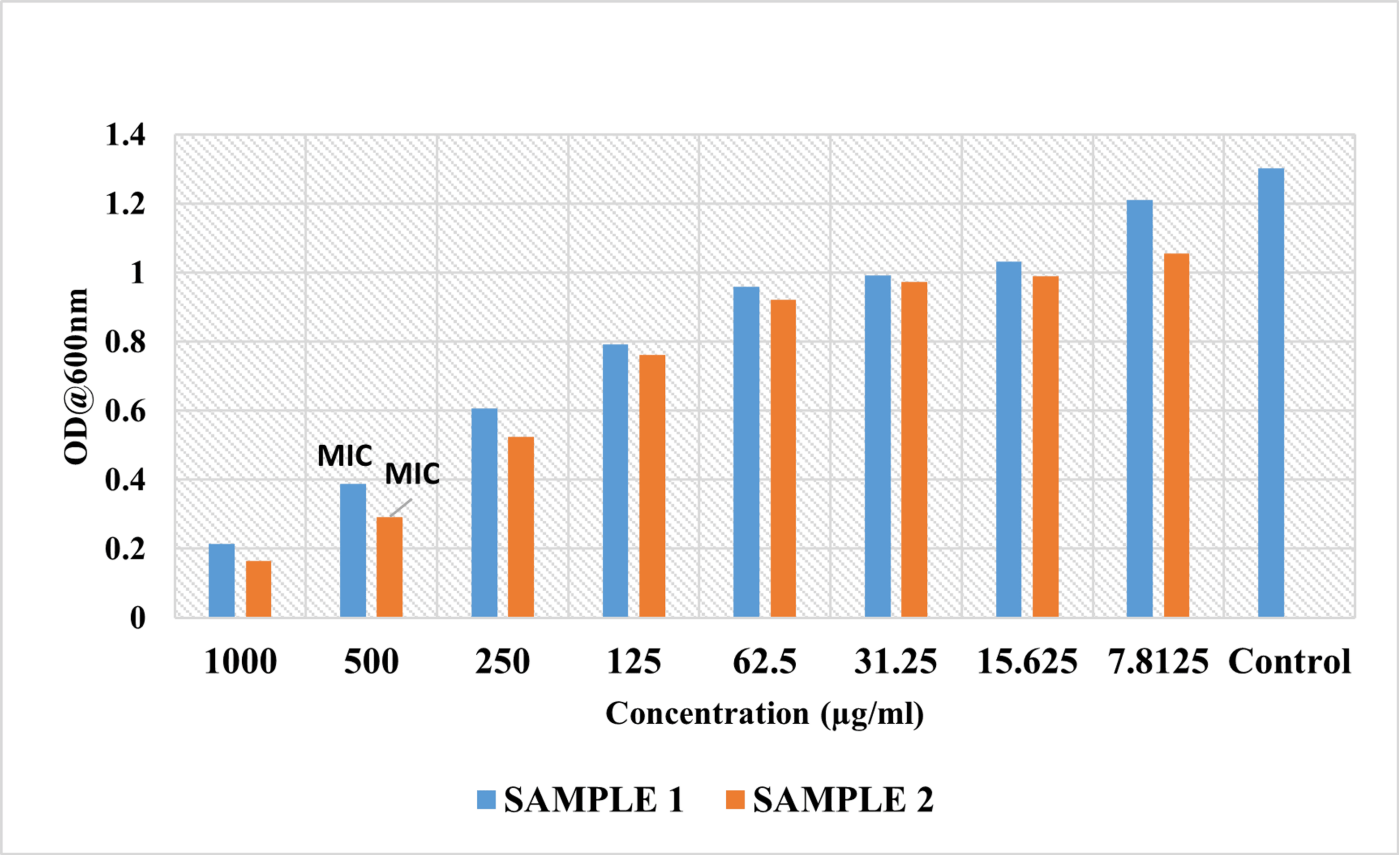
Minimum inhibitory concentration of aqueous and ethanolic extract of Cissus quadrangularis Sample 1: Aqueous extract of *Cissus quadrangularis.* Sample 2: Ethanolic extract of* Cissus quadrangularis* dissolved in 10% DMSO. MIC: minimum inhibitory concentration, DMSO: dimethyl sulfoxide, OD: optical density.

**Figure 5:**
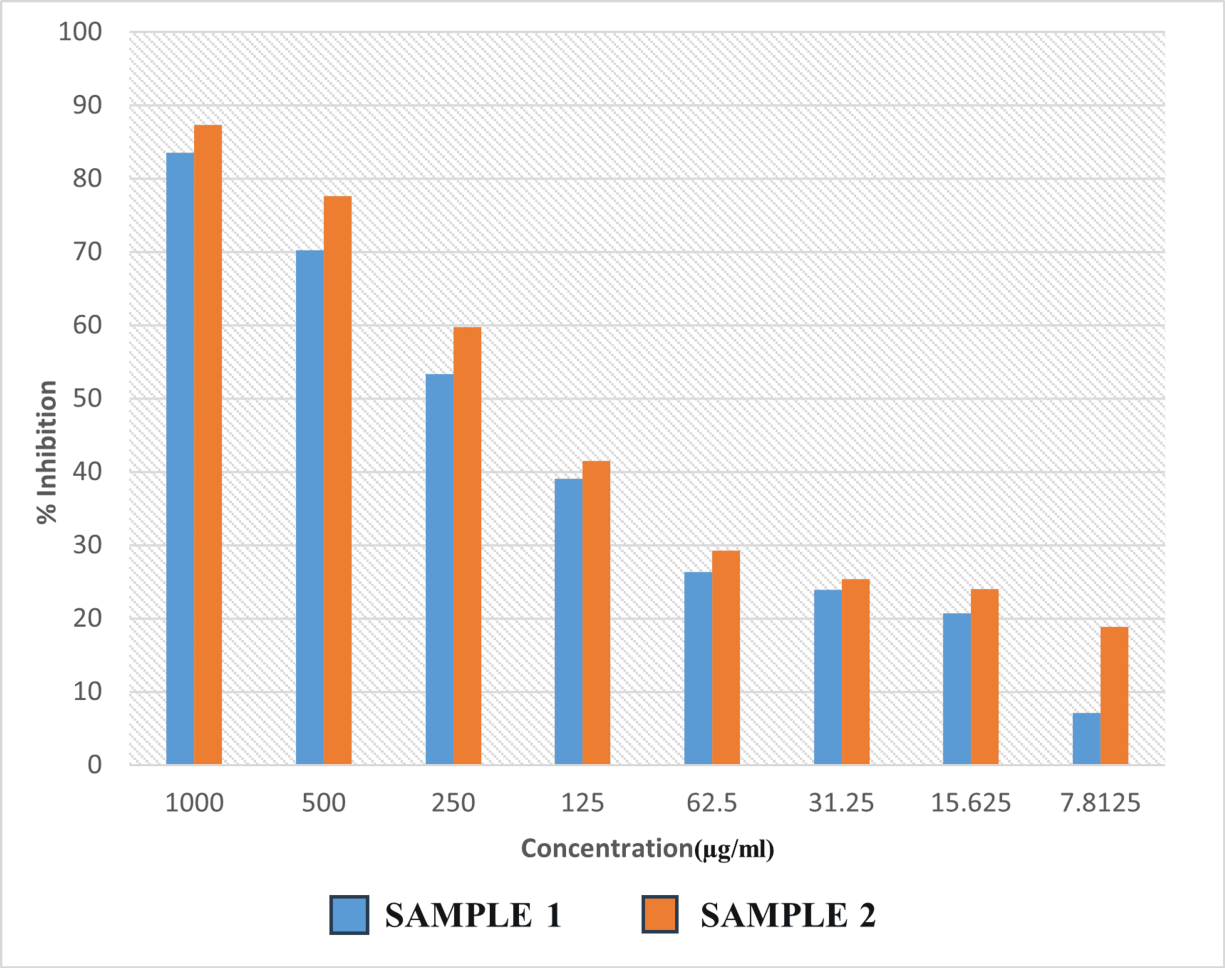
Percentage inhibition of aqueous and ethanolic extract of Cissus quadrangularis Sample 1: Aqueous extract of *Cissus quadrangularis.* Sample 2: Ethanolic extract of *Cissus quadrangularis* dissolved in 10% DMSO. DMSO: dimethyl sulfoxide.

The MIC data reveal intriguing insights into the inhibitory potential of Sample 1 (an aqueous extract of CQ) and Sample 2 (an ethanolic extract of CQ dissolved in 10% DMSO) at varying concentrations. The concentrations tested ranged from 1000 µg/mL to 7.8125 µg/mL. The MIC, representing the minimum concentration at which microbiological development is prevented, is a critical parameter in assessing the effectiveness of antimicrobial agents. The MIC of Samples 1 and 2 was determined to be 500 µg/mL, with inhibitory percentages of 70.19% and 77.57%, respectively (Table 1 and Figure 5). As per standard protocol, the IC50 of Samples 1 and 2 was determined to be 240.46 µg/mL and 194.36 µg/mL, respectively. The inhibitory effect of the ethanolic extract of CQ dissolved in 10% DMSO was better than that of the aqueous extract of CQ. The broth microdilution method illustrated in Figure 3 provides a visual representation of the antimicrobial activity of the samples, highlighting their ability to inhibit bacterial growth at varying concentrations. Furthermore, Figure 4 depicts the activity of the samples compared to the control in terms of optical density (600 nm), reaffirming their effectiveness in suppressing bacterial proliferation.

**Table 1:** Inhibitory Percentage and IC50

Concentration (µg/mL)	Sample 1	Sample 1 - IC50	Sample 2	Sample 2 - IC50
1000	83.56	240.46 µg/mL	87.33	194.36 µg/mL
500	70.19		77.57	
250	53.37		59.75	
125	39.09		41.47	
62.5	26.34		29.26	
31.25	23.89		25.34	
15.63	20.74		24.04	
7.81	7.07		18.89	

## Discussion

Gingivitis and periodontitis are prevalent periodontal disorders that impact a substantial proportion of the worldwide population. These chronic inflammatory diseases are predominantly caused by an excess of certain anaerobic bacterial species in dental plaque. This is usually treated with mechanical methods (scaling and root planing, or SRP), but these methods are not always successful because of pathogens that can invade tissue. Systemic and local antibiotics may be beneficial when used in conjunction with mechanical treatment. However, widespread antibiotic use can lead to antibiotic resistance. As a result, research over the past ten years has concentrated on medicinal plants with anti-inflammatory and antibacterial properties to treat these ailments.

The foundations of traditional medicinal systems have historically been plants [23]. Plant extracts, in particular, have emerged as promising natural antibacterial agents with diverse therapeutic applications for combating pathogenic bacteria. CQ, for instance, contains a plethora of beneficial compounds such as carotene, phytosterol, terpenoids, β-sitosterol, δ-amyrin, δ-amyrone, calcium, flavonoids like quercetin, vitamin C, and stilbene derivatives such as resveratrol, piceatannol, pallidol, perthenocissin, and phytosterols [15].

The aqueous and ethanolic extracts of CQ were selected for the current investigation in order to assess their antibacterial activity against *P. gingivalis*, the keystone periodontal pathogen. The results of our study shed light on the inhibitory potential of two different samples of CQ aqueous and ethanolic extracts against *P. gingivalis*, a prominent periodontal pathogen. The MIC data reveal the inhibitory potential of Sample 1 (an aqueous extract of CQ) and Sample 2 (an ethanolic extract of CQ dissolved in 10% DMSO) at varying concentrations. The concentrations tested ranged from 1000 µg/mL to 7.8125 µg/mL. The MIC of Samples 1 and 2 was determined to be 500 µg/mL. Additionally, the inhibitory percentages of Sample 1 and Sample 2 were found to be 70.19% and 77.57%, respectively.

The determination of the half-maximal inhibitory concentration (IC50) further elucidated the potency of the samples, with Sample 2 exhibiting a lower IC50 value (194.36 µg/mL) compared to Sample 1 (240.46 µg/mL). These findings indicate that the ethanolic extract of CQ formulated with 10% DMSO possessed enhanced antibacterial activity against *P. gingivalis* compared to the aqueous extract (Sample 1). The results were consistent with the findings of Nadhini et al., who reported that the ethanolic extract of CQ exhibited the highest level of antibacterial activity against clinical pathogens [24]. They stated that the ethanolic extract was most frequently used and showed a reasonable antibacterial effect.

Multiple clinical investigations have indicated that administering CQ within a range of 1.2-10 g/day for durations spanning 4-12 weeks led to an acceleration in bone fracture healing, without encountering severe adverse events (AEs). These clinical observations find reinforcement in various in vivo and in vitro studies focusing on bone-related outcomes. Such collective evidence underscores the potential of CQ as an alternative approach to mitigating bone loss [25,26].

The traditional medicinal practice of Ayurveda has long utilized CQ to address a spectrum of diseases and ailments [27]. Its recognized osteogenic properties prompted an exploration into its potential application for periodontitis. Our findings confirm the antibacterial properties of CQ, shedding light on its potential therapeutic role in combating periodontal infections. The efficacy of both samples in preventing the growth of *P. gingivalis* is of significant clinical relevance, as it suggests their potential utility in the prevention, treatment, and management of periodontal diseases.

The strength of our study lies in its novel focus on the antibacterial activity of CQ against *P. gingivalis*, a key pathogen in periodontal disease. The demonstrated efficacy of CQ, particularly the ethanolic extract, suggests its potential as an adjunctive therapy for periodontal diseases. Furthermore, incorporating CQ into local drug delivery systems, such as mouth rinses and gels, could enhance its therapeutic application in dental practice.

Despite the promising results, the study is subject to certain limitations. While the extracts effectively targeted *P. gingivalis*, their efficacy against other periodontal pathogens remains unknown due to the exclusive focus on this single bacterium. Furthermore, the controlled laboratory environment (in vitro) necessitates clinical trials to validate their effectiveness for further clinical applications.

## Conclusions

In conclusion, our findings highlight the significant antibacterial properties of CQ aqueous and ethanolic extracts against *Porphyromonas gingivalis*. The demonstrated efficacy of CQ extracts in inhibiting *P. gingivalis* opens new avenues for the development of innovative periodontal treatment strategies. To fully harness the potential of CQ extracts, further research is necessary to delve into the underlying mechanisms of their antibacterial action. Understanding these mechanisms will provide valuable insights into how these extracts can effectively combat *P. gingivalis .*
